# Determination of Thermoluminescence Kinetic Parameters of La_2_O_3_ Doped with Dy^3+^ and Eu^3+^

**DOI:** 10.3390/ma13051047

**Published:** 2020-02-26

**Authors:** Mahmoud Bakr, Mohamed Omer

**Affiliations:** 1 Institute of Advanced Energy, Kyoto University, Uji, Kyoto 611-0011, Japan; 2 Physics Department, Faculty of Science, Assiut University, Assiut 71516, Egypt

**Keywords:** thermoluminescence, kinetic parameters, different heating rates, activation energy, three-points analysis method, La_2_O_3_ nanophosphors

## Abstract

Thermoluminescence (TL) properties of La_2_O_3_: Dy^3+^, Li^+^, and La_2_O_3_: Eu^3+^, Li^+^, exposed to 5.12 Gy of beta radiation, and recorded at different heating rates 0.5, 1, 2, 3, 4, and 5 °C s^−1^ (from Molefe et al., paper 2019), were analyzed and the trap parameters were determined in this study. These parameters include the order of kinetics *b*, the activation energy *E* (eV), the frequency factor *S* (s^−1^), or the pre-exponential factor *S*″ (s^−1^), and the initial concentration of trapped electrons *n_o_* (cm^−3^). A new non-linear curve fitting technique, based on the general order kinetic equation and the outcomes of Hoogenstraaten’s Method, was established and applied on the TL glow peaks of La_2_O_3_: Dy^3+^, Li^+^. The fitting technique was evaluated by calculating the R-square and figure of merit (FOM) values. The results revealed that the FOM values are <1%, and the R-square values are >0.997, which demonstrates an excellent convergence between experimental and fitted curves. A modified technique based on the three-points analysis method was exploited to deconvolute complex TL glow curves of La_2_O_3_: Eu^3+^, Li^+^, and in turn, to determine the trap parameters the method disclosed that each TL glow curve consists of four peaks. The trap parameters of the individual peaks were numerically determined. The fading, as a function of storage temperature and time, from the TL signals of the investigated materials was predicted and discussed based on the calculated trap parameters. The results support the value of the materials for employment in radiation dosimeter applications with a low fading fraction.

## 1. Introduction

Thermoluminescence (TL) is the emission of light through heating a phosphor material after absorbing energy from ionizing radiation [1]. The released energy, in the form of luminescence, and sensitivity of photon detection make the TL phenomenon an attractive method to measure small quantities of stored energy. The development of luminescent materials has been successfully applied in diverse fields and has dramatically enhanced human life [2]. Applications of the TL phenomenon are found not only in dosimetry, research, earth sciences, and the age determination of archaeological or geological samples [3,4,5,6,7], but also in light-emitting diodes for home lightning [8], and bio-imaging medicine for cancer cells mapping [9].

TL is a beneficial method to study the interaction of radiation with the defects inside the material lattice. This takes place through monitoring the movement of the electrons between the trap and recombination centers. These movements can be straightforward, such as one process of electron release from the trap and then recombine into a recombination center, or intricate. That includes several events such as releasing, trapping, escaping, tunneling and recombining of electrons through the trap and recombination centers before emitting a light [10]. The intensity of the emitted light is plotted as a function of the heating temperature to produce a TL glow curve, which is a fingerprint property for each material. The TL curve is used to determine the kinetic parameters of the trap centers inside the lattice. Based on the number of trap bands in the material, the glow curve appears.

Several models have been applied to describe the TL process that arose in materials. The easiest one, named first order kinetics, assumes a thermal release of an electron from trap centers is followed by recombining with a hole in the recombination centers. In this model, the probability of the electron being retrapped is assumed to be impossible [11]. While the second order model considers two possibilities are granted to the electrons upon the thermal release: recombine with holes or re-trap again [12]. An empirical model has been suggested to describe the situation when the TL peak of neither first order nor second order are satisfied, named general order kinetics [13]. Specific details of the trap and recombination centers provide valuable information about the material properties, defects, and history of radiation absorbed. This information is found in the trap parameters of the centers. The parameters include the depth of trap center (activation energy) *E* (eV), the kinetic order of TL processes *b* (first, second and general), the initial concentrations of trapped charges *n*_0_ (cm^−3^), the frequency factor *S* (s^−1^), (probability of escaping) in the case of first order or pre-exponential factor *S*″ (s^−1^) in the case of second and general order kinetics. The TL glow curves are usually analyzed by assuming different *b* values, i.e., assume the first, second, or general order model.

Several methods have been developed and applied to obtain the trap parameters of a single TL peak [1,3,5,10,14,15,16,17,18,19,20,21,22,23]; however the number is dwindling in the case of overlapped peaks [24,25,26,27,28,29]. The methods proposed to determine the trap parameters must take into consideration the effects of overlapped peaks. Therefore, the main obstacle for obtaining the trap parameters is the presence of several overlapped peaks within the TL glow curve. Very few methods exist for the separation of the glow curve into its components of glow peaks [24,25,26,27,28,29]. In order to obtain the physical parameters associated with various TL bands, it is necessary to fit a theoretical equation containing these parameters to the entire glow curve [22]. Generally, if the experimental TL glow curves are straightforward obeying one of the standard models, first or second model, the usage of the general order equation to do analysis for the data has no meaning. Even with the fact that the obtained results will give approximately the same values estimated from the first and second orders.

The radiation dose and the heating rate strongly affect the shape and position of TL glow peaks inside the glow curve. The theory predicts that increasing the TL intensity while the peak position is kept constant is due to an increase in the radiation dose. On the other hand, a linear increase in the heating rate results in a drastic decrease in the TL intensity and shifts the peak position to the higher temperature values [1]. Several authors have proposed and applied methods to calculate *E* by subjecting the material to various heating rates (VHR) [30,31,32,33,34,35,36]. These methods are predominantly applicable for a single glow peak, and some used the methods to guess the value of *S*. However, if the glow curve is composed of overlapped peaks, it is difficult to trust these methods to determine *E* and *S* with reasonable accuracy.

The quality of the luminescent materials, which is adapted for radiation dosimeter applications, is measured with the constancy of their TL signal against the fading [3]. The fading of the TL sample is evaluated by comparing the recorded TL signals before and after the storage under fixed environmental conditions. The proper material for the radiation dosimeter application is the smaller fading. An inefficient and forthright way to predict the tendency of the material against the fading is to determine the decay of the signal by use of equations include the trap parameters of the material.

The central aim of the present work is to determine the trap parameters, including *b*, *E*, *n*_0_, and *S* or *S*″, of La_2_O_3_: Dy^3+^, Li^+^, and La_2_O_3_: Eu^3+^, Li^+^ recorded at different heating rates. Additionally, to evaluate the suitability of the investigated materials as candidates for radiation dosimeter application by studying the fading of the TL signal as a function of the storage conditions. The TL glow curve from La_2_O_3_: Dy^3+^, Li^+^ material shows a single glow peak, while La_2_O_3:_ Eu^3+^, Li^+^ shows a complex glow curve. Two different methods are used in this study to analyze the TL glow curves and determine the trap parameters of the materials. A new, non-linear curve fitting program, based on the general order kinetic equation and the results from Hoogenstraaten’s Method, is suggested and applied for the single glow peaks. A modified version of the three-points analysis method is applied to separate and determine the trap parameters of the complex glow curves. Eventually, the fading from the investigated materials is predicted using the decay equations of the TL signal as a function of storage conditions. The trap parameters, obtained for the investigated materials, are used to predict and discuss the influence of the storage conditions on the material fading.

## 2. Methodology

### 2.1. Mathematical Treatment

The first model for the TL phenomenon is ascribed to Randall and Wilkins [11]. Considering a simple process of thermal release of electrons from traps and their subsequent recombination with holes in recombination centers, with no possibility of retrapping. The TL intensity from the first order model is given as [11]:

(1) I(T)=noSexp(−EkT)exp[−Sβ∫T0Texp(−EkT′)dT′]

where *I* (in arbitrary units) is the TL intensity, *T*_0_ (K) is the starting heating temperature, *β* (°C s^−1^) is the heating rate, and *k* (eV/K) is Boltzmann’s constant. By applying the first derivative, concern the temperature, on Equation (1) and then equating it to zero, the position of maximum TL intensity (*I_m_*) can be determined as:

(2) βEkTm2=Sexp(−EkTm)

where *T_m_* is the position corresponding to *I_m_* of the peak. Arranging Equation (2) gives the value of *S*:

(3) S=βEkTm2exp(EkTm)

The TL intensity for the second order kinetics is given by Garlick and Gibson in the form [12]:

(4) I(T)=n02Sexp(−EkTm)N[1+(n0SβN)∫T0Texp(−EkT′)dT′]2

where *N* (cm^−3^) is the traps concentration.

Finally, the TL intensity in the case of general order kinetics, which is the most utilized for analyzing TL glow curves, is given in its final form for *b* ≠ 1 as [13]:

(5) I(T)=noS″exp(−EkTm)[1+(b−1)S″/β∫T0Texp(−EkT′)dT′]b/(b−1)

where *S*″ = *S*(*n_o_*/*N*)^*b*−1^, *b* is the parameter related to the retrapping probability. Note that when approaching 1, Equation (5) converges to Equation (1), first order, and when *b* approaching 2, converges to Equation (4), second order kinetics. Taking the first derivative of the TL intensity of Equation (5), concerning the temperature, and equating to zero results in the condition of a maximum for general order kinetic as:

(6) 1+(b−1)(S″β)ϕ=(S″bkTm2βE)exp(−EkTm)

where

(7) ϕ=∫T0Texp(−EkT′)dT′

The next subsections are used to give the theoretical background of the techniques used in the study.

#### 2.1.1. Hoogenstraaten’s Method

Hoogenstraaten used Equation (2) at VHR to obtain different glow peaks in the first order model to estimate the relation between the *E* and the heating rate as follows [33]:

(8) $$\ln\left(\frac{T_{m}^{2}}{\beta }\right)=\left(\frac{E}{K}\right)\frac{1}{T_{m}}-\ln\left(\frac{kS}{E}\right)$$

Thus, plotting ln(*T*^2^*_m_*/*β*) with (1/*T_m_*), giving rise of a straight line, from the slope the value of *E* can be determined, and from the intercept, the value of *S* can be calculated. Despite the fact this method has been developed for the first order kinetics, many authors [1,5,6,7,23,34] have shown that Equation (8) can be used to obtain *E* as an acceptable approximation for non-first order situations. In the case of general order kinetics, the term ln(*kS*/*E*) is replaced with ln(*kS*″/*E*) then Equation (8) is written in the adapted form as [23]:

(9) $$\ln\left(\frac{T_{m}^{2}}{\beta }\right)=\left(\frac{E}{K}\right)\frac{1}{T_{m}}-\ln\left(\frac{kS\prime \prime }{E}\right)$$

From the slope and intercept of Equation (9), the values of *E* and *S*″ can be estimated. The parameters *b* and *n*_0_ then can be obtained afterward by fitting *E* and *S*″ over an experimental glow curve using a curve fitting program.

Fitting programs are suitable methods to analyze the TL glow curves. It has been used for a decade with excellent precision, especially to analyze single peaks using the first order model [37]. The most important fitting methods are based on the first order kinetics model or Gaussian functions. The initial has achieved considerable success to fit experimental curves to the model of the first order. The main disadvantage of these methods appears in the inadequacy to fit a non-first order glow peaks [38]. The symmetric Gaussian fitting functions are possibly applicable to fit the second order kinetics. That mainly used for dosimetry analysis; if the order of kinetics is known in advance, also when calculating the integral of the peak is more critical than determining the trap parameters. The drawback of this method is that the function is not based on a physical model. Thus, it is improper to determine the order of kinetics for non-second order. Accordingly, to calculate other trap parameters is not truthful.

A suitable analysis method, using the general order equation, represents an urgent demand in the TL research and application fields. The general order equation is the most widely applicable equation for describing the TL glow curves. It has advantages over the first order kinetics and Gaussian function methods, such as the possibility to determine the value of *b*, calculate all trap parameters included in the general order equation, and the influence of the parameters on each other are considered. On the other hand, fitting using Equation (5) is more complicated compared to other methods since one more parameter, *b*, for each peak needs to be fitted. Moreover, the typical difficulty of using Equation (5) is how to calculate the integral appeared in Equation (7). Haake has established an approximation for the integral in asymptotic series in the form [39]:

(10) ∫T0Texp(−EkT′)dT′=Texp(−EkT)∑1m(kTE)m(−1)m−1 m!

where *m* is the number of terms of the asymptotic series, which defines the accuracy of the approximation. The basic terms for a good approximation of the integral are two terms [37]. In the present study, four terms are considered through the fitting process. By inserting the approximation of the integral in Equation (5), one obtains:

(11) $$I\left(T\right)=n_{o}S\prime \prime \exp\left(-\frac{E}{kT}\right){\left[1+\left[\left(b-1\right)\frac{s\prime \prime }{\beta }\right]\left(\frac{kT^{2}}{E}\right)\exp\left(-\frac{E}{kT}\right)\left(1-2\left(\frac{kT}{E}\right)+6{\left(\frac{kT}{E}\right)}^{2}-24{\left(\frac{kT}{E}\right)}^{3}\right)\right]}^{-b/\left(b-1\right)}$$

Equation (11) will be used to fit a TL glow curve and calculate the values of *b* and *n*_0_ by the use of a non-linear curve fitting program. This program is analyzed utilizing the ORIGIN package, herein O-CFP. The values of *E* and *S*″, determined from Equation (9), in addition to the *β,* are the primary input for the program, while an initial guess for *b* and *n*_0_ are supplied as a guide. In order to increase the accuracy of the fitting and get reasonable values of the fitted parameters, at least two initial values should be provided, invariable parameters, to the program, such as (*E*, *β*), (*S*″, *β*), or (*E*, *S*″). To speed up the convergence, which means reducing the number of iterations, the values of (*E*, *S*″, *β*) are needed as constant parameters for the program.

The quality of the fitting is estimated using the figure of merit (FOM) equation, which is given from [40]:

(12) $$\mathrm{FOM}\%=\frac{\sum_{i}\left|Y_{i}-F_{i}\right|}{\sum_{i}Y_{i}}\times 100$$

where *Y_i_* is the input value (experimental or numerical data), and *F_i_* is the best-fitting value of the TL intensity at temperature *T_i_*. The lower the FOM, the better the quality of fitting. The ideal value of FOM is 0, which is obtained only when the fit is perfect. However, values less than 1% may be considered reasonably good, assuming several experimental and theoretical errors in the glow curve measurements and fitting, respectively [41,42,43]. FOM values greater than 2% implies significant errors in the fitted values. Furthermore, the quality of the fitting in the O-CFP has been evaluated by calculating R-square values to specify how close the data are to the fitted curve. Evaluation of the O-CFP for fitting glow peaks and obtaining *b* and *n*_0_ is demonstrated by taking a numerically generated TL glow peaks (see Appendix A). However, the applicability of the method is demonstrated here by taking experimental TL glow curves at different heating rates of La_2_O_3_: Dy^3+^, Li^+^ [36].

Five or more different heating rates are obligatory to get a trusted fitting and determine accurate values of *E* and *S*″ using Hoogenstraaten’s Method, which could be one of the obstacles in front of the present method. Nonetheless, providing non-accurate values of *E* and *S*″ to the O-CFP program leads to an overestimate in the fitted values of *b* and *n*_0_. Moreover, the uncertainty of determining the values of *T_m_* could represent an additional difficulty using the present method.

#### 2.1.2. Basics of the Three-Points Analysis Method

The three-points analysis (TBA) method is a technique to deconvolute a complex glow curve and determines the trap parameters, including *b*, *E*, *n*_0_, and *S* or *S*″, of the individual peaks. It has been proposed for the first time by Rasheedy [26]. The technique successfully used to perform analysis for TL glow curves consisted of two and five glow peaks [44,45]. The TPA technique starts with selecting a set of three points, *x*, *y*, and *z*. In other words, three temperatures, such as *T_x_*, *T_y_*, and *T_z_*. The intensities of the points in the glow peak are *I_x_*, *I_y_*, and *I_z_*, respectively. The points are randomly selected through the glow peak, considering the overlapping conditions with *y = (I_x_/I_y_)* and *z = (I_x_/I_z_)*. The values of *A_x_*, *A_y_*, and *A_z_* are the areas under the peak between the temperatures *T_i_* and the final temperature of the glow peak *T_f_*. By defining the above values, the value of *b* can be determined numerically using the form [26]:

(13) b=Ty[Tx−Tz]ln(y)−Tz[Tx−Ty]ln(z)Ty[Tx−Tz]ln(AxAy)−Tz[Tx−Ty]ln(AxAz)

In sequence, the activation energy can be calculated from the expression:

(14) E=[ln(y)−bln(AxAy)](kTxTyTx−Ty)

or from the expression:

(15) E=[ln(z)−bln(AxAz)](kTxTzTx−Tz)

Then, the value of *S* in the case of first order kinetics is calculated from:

(16) S=(βEkTm2)exp(EkTm)

Alternatively, *S*″ in case of the general order can be obtained as:

(17) S″=βEexp(EkTm)[bkTm2]−(b−1)Eϕexp(EkTm)

where *ϕ* has the same definition given in Equation (7). Eventually, the relative concentration of the charge carriers *n*_0_ can be estimated from the following expression:

(18) n0=Imexp(EkTm)S″[bkTm2S″βEexp(EkTm)]b/(b−1)

Benefits of the TPA technique are: (i) only an experimental TL curve and *β* value are needed to start the method; (ii) no need for pre-calculations of any of the trap parameters or earlier information about the TL glow curve; (iii) all the trap parameters are calculated sequentially using Equations (13)–(18); (iv) the technique is not only used to determine the trap parameters of the glow peak but also to separate the overlapped glow curve into its components of glow peaks. The primary mathematical treatment of the technique and the derivation of the equations mentioned above are given in detail elsewhere [40]. The TPA technique is applied here to analyze and obtain the trap parameters of the experimental TL glow curves of La_2_O_3_: Eu^3+^, Li^+^ after being exposed to 5.12 Gy beta radiation, and recorded at *β* = 0.5, 1, 2, 3, 4, and 5 °C s^−1^ [36].

One of the present method limitations is that the method is not applicable to a non-ending glow curve as the area under the curve between *T_i_* and *T_f_* is calculated in the TPA program sequence, and the results are strongly affected by the values of *A_i_*. In addition, one of the drawbacks of the method is that it cannot be used for very complex TL glow curves when peaks interlay or overlap.

#### 2.1.3. Fading of the TL Signal

Radiation dosimeters, both personal and environmental, are widely used in the fields of nuclear power production, medicine, and research applications [7,46,47]. Generally, the material stores the received radiation in the form of energy stored by reordering the position of the charge carriers in the lattice for the accumulation period. Then, the information about the dose is collected by readout the signal from the device (c.f. heating the material to emit TL glow curve). A fraction of the stored energy in the lattice, unfortunate, is fading before being recorded, especially when the temperature changes significantly during the accumulation. The time between receiving the radiation dose and read the signal has a robust influence on the acquired signal. Besides, the environmental conditions during the accumulation, such as the temperature, are altering the final signal. Indeed, information about the collected does with high accuracy is one of the challenges for the material used in this filed.

The fading of the TL signal from the material is a limiting factor for such long-term applications as a dosimeter device. Therefore, identifying the fading fraction of the TL material is an essential step through the selection of the material. The fading of the stored dose takes place due to the escape of the electrons from the trap centers. As a result, a change of the electrons distribution, the population of the centers inside the lattice, most likely change. That occurs in the form of migration and aggregation of the electrons [7]. The predominant factor that influences the fading is the storage temperature, while the storage time and the kinetics of the charges inside the lattice also affect the process. Indeed, to ascertain the kinetics of the charges is to determine the trap parameters for the centers inside the lattice. The stability of the TL signal against the fading is considered an essential property for the TL material planned to be used in radiation dosimeter applications. Irradiate the samples and read out the TL signal at different time intervals, such as hours, days, weeks, or months, and compare the signals with that at *t*_0_, immediately after irradiation, is the usual method for studying the fading of the TL signal. The small fraction of the faded signal means the high quality of the material to store the information at a constant temperature.

In the present study, the faded fraction of the TL signals of La_2_O_3_: Dy^3+^, Li^+^, and La_2_O_3_: Eu^3+^, Li^+^ is estimated based on the trap parameters information. The connection between the faded signal and the original at *t*_0_ can be expected from the equations that describe the order kinetics models. The rate of release electrons per unit time at a temperature *T* of the first order kinetics is given in the form [11]:

(19) $$-\frac{dn}{dt}=n\;S\;\exp\left(-\frac{E}{kT}\right)$$

where *n* (cm^−3^) is the concentration of electrons at time *t*(s). If the material is irradiated and then stored at a fixed temperature for time *t*, Equation (19) can be rearranged as:

(20) $$-\frac{dn}{n}=S\;\exp\left(-\frac{E}{kT}\right)dt$$

The integration of Equation (20) can provide the decay of the TL signal as a function of the storage time at a given constant temperature as:

(21) $$n\left(t\right)=n_{0}\;exp\left(-S\;t\exp\left(-\frac{E}{kT}\right)\right)$$

where *n*_0_ is the initial concentrations of the electrons at *t*_0_. Similarly, the decay of the TL signal in the case of the second order kinetics can be given in the form [12]:

(22) $$n\left(t\right)=n_{0}\;{\left[1+n_{0}\left(\frac{S}{N}\right)t\exp\left(-\frac{E}{kT}\right)\right]}^{-1}$$

Eventually, the decay of TL signal for the general order kinetics is given as:

(23) $$n\left(t\right)=n_{0}\;{\left[1+S{\left(\frac{n_{0}}{N}\right)}^{b-1}t\left(b-1\right)\;\exp\left(-\frac{E}{kT}\right)\right]}^{1/\left(1-b\right)}$$

where *S*″ = *S*(*n*_0_/*N*) ^*b*−1^ and *b* have the same definition given above. In Equation (23) by inserting *b* = 2, it gives the TL decay of second order, Equation (22). Although Equation (23) is not valid for the case *b* = 1, it can be reduced to Equation (21) when *b* approaching 1 [13,37,48]. Now, if the trap parameters of the glow peak are given, one can use Equations (21)–(23) to estimate the behavior of the first, second, and general order TL glow peaks, respectively, against the signal fading.

### 2.2. TL Glow Curves Used for the Present Analysis

**Figure 1:**
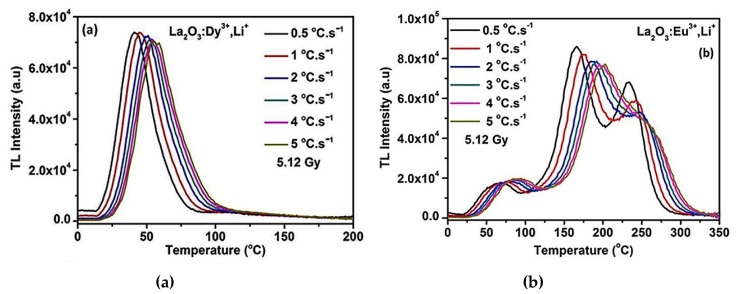
The TL glow curves obtained at *β*= 0.5, 1, 2, 3, 4, and 5 °C s^−1^ for (**a**) La_2_O_3_: Dy^3+^, Li^+,^ and (**b**) La_2_O_3_: Eu^3+^, Li^+^ after exposed to 5.12 Gy of beta radiation, reproduced with permission from [36]. Copyright 2019 Elsevier.

Lanthanum Oxide (La_2_O_3_) is a semiconductor with a 5.8 eV bandgap. It has unique optical and magnetic properties due to the 4*f* level configuration [46]. Doping La_2_O_3_, according to density functional theory, is expected to significantly change the characteristics of the material as energy storage [47,48]. Dy^3+^ and Eu^3+^ were used to activate La_2_O_3_ in order to attain proper luminescence characteristics. The microwave-assisted solution combustion method was used to co-activation La_2_O_3_ with Li^+^. The nanophosphors of La_2_O_3_: Dy^3+^, Li^+^, and La_2_O_3_: Eu^3+^, Li^+^ were exposed to 5.12 Gy beta radiation. Then, the TL glow curves from the materials were recorded at different heating rates 0.5, 1, 2, 3, 4, and 5 °C s^−1^. The glow curves of La_2_O_3_: Dy^3+^, Li^+^, and La_2_O_3_: Eu^3+^, Li^+^ are presented in Figure 1a,b, respectively. The data points of the glow curves were picked up from the figures in [36] by digitizing the data points, and then the data were interpolated.

## 3. Results and Discussion

### 3.1. Applications of Hoogenstraaten’s Method and O-CFP for Obtaining the Trap Parameters of La_2_O_3_: Dy^3+^, Li^+^

The primary aim of this part is to determine the values of *E* and *S*″ of the TL glow curves of the La_2_O_3_: Dy^3+^, Li^+^ shown in Figure 1a, by applying the Hoogenstraaten’s Method. Then apply the suggested fitting program, O-CFP, to determine *b* and *n*_0_. From Figure 1a, the TL glow curves appear in a single well-defined glow peak. The values of heating rates *β* and corresponding maximum temperatures *T_m_* for these glow curves are obtained and listed in Table 1. Two indicators are shown in Figure 1a as a direct result of increasing the heating rate: (i) the peak position is shifted towered the high-temperature values and (ii) the TL intensity decreases with increasing the heating rate, which may be explained by the thermal quenching effect [48,49]. Indeed, the observed effect of the heating rate on the TL glow curves is expected, and the material is obeying the theoretical predictions.

**Figure 2:**
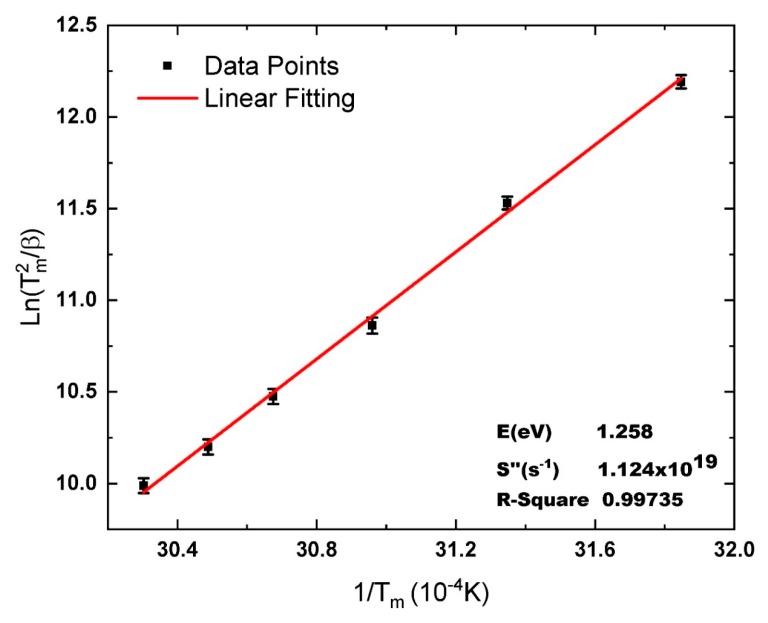
Hoogenstraaten’s Method plotted according to Equation (9) for data shown in Table 1.

The data listed in Table 1 are plotted according to the adapted Hoogenstraaten’s Method, Equation (9). The relation between ln*(T^2^_m_/β)* and *(1/T_m_)*, giving rise to a straight line, as shown in Figure 2. From the slope (*E*/*k*) and the intercept ln*(kS″/E)*, the values of *E* and *S*″ can be calculated. The activation energy *E* is found equal to 1.258 eV, and from the intercept, the value of the pre-exponential factor *S*″ is 1.124 × 10^19^ s^−1^. The R-square value, to evaluate the quality of the fitting, is found to be 0.99735. The value of *E*, estimated from the present method is matching very well with the other techniques, such as (i) the initial rise method by considering up to 15% of the low-temperature side of the peak [46]; (ii) and VHR method with the approximated value of *E* = ln(*T*^2^*_m_*/*β*) *kT_m_*, [47,48]. The value of *E* is found equal to 1.25 ± 0.02 eV from the first method and 1.31 ± 0.02 eV from the second [36]. The advantage of using the adapted Hoogenstraaten’s Method is the possibility to obtain *S*″. Thus, it is considered a step forward to decrease the number of unknown parameters for the fitting program. The values of *E* and *S*″ with the heating rate are used in the second part of this section for fitting the experimental TL glow curves, to the general order kinetics equation, using the O-CFP and then determine the values of *b* and *n*_0_. For simplicity, detailed explanations of fitting the TL curve of La_2_O_3_: Dy^3+^, Li^+^ recorded at *β* = 2 °C s^−1^, are illustrated in this section. The rest of the TL glow curves are identically treated, and only concluding results are given.

**Figure 3:**
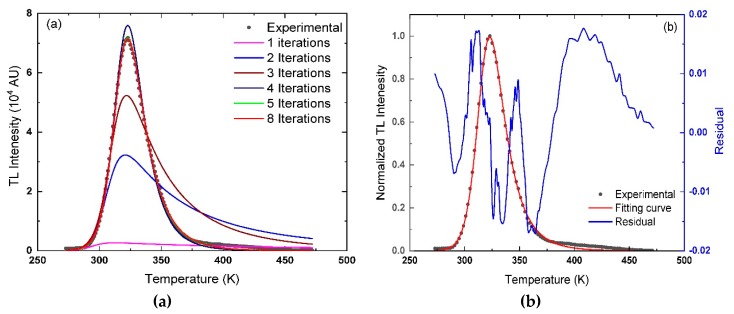
(**a**) The growth of the theoretical curves as a function of iterations number compared with the experimental curve of La_2_O_3_: Dy^3+^, Li^+^, at *β* = 2 °C s^−1^, (**b**) the normalized experimental and fitted curves with the residual from the fitting process.

The sequence in the O-CFP starts with inserting Equation (11) into the ORIGIN package. The equation is used to simulate a theoretical curve based on the constraints and the input parameters given to the program. The values of *E* = 1.258 eV, *S*″ = 1.124 × 10^19^ s^−1^, and *β* = 2 °C s^−1^, invariable parameters for the program, are the input parameters. The next step is to suggest the first guess for *b* and *n*_0_. The first guess of *b* and *n*_0_, in this case, are 1.001 and 1000 cm^−3^, respectively. Then, the values of *b*, *n*_0_, *E*, *S*″ and *β* are used in Equation (11) to simulate a theoretical curve. The simulated curve, using the given parameters, is shown in Figure 3a and named as the first iteration. Then, the program assesses the theoretical curve in comparison with the experimental one.

Consider R-square, FOM, and other constraints, the direction to optimize the fitting, and then the O-CFP suggests the second guess. The shape of the peak generated using the first guess is very far from the experimental curve, as seen in Figure 3a. In order to achieve convergence between the experimental and the simulated curves, the program is performing a change in *b* and *n*_0_ values. The new values of *b* and *n*_0_, with *E*, *S*″ and *β,* are used to generate a theoretical curve, second iteration in Figure 3a. The O-CFP iterations continue until convergence between experimental and simulated curves takes place. That occurs by looking into the values of R-square, FOM, and the residual of the peak, the difference between the experimental and fitted curves. Figure 3a shows the growth of the fitted curves as a function of the iteration number for experimental TL peak obtained at *β* = 2 °C s^−1^. In addition, Figure 3b shows the normalized experimental and fitted curves for La_2_O_3_: Dy^3+^, Li^+^ glow curve recorded at *β* = 2 °C s^−1^ with the residual curve.

Here, it is found that the best values of *b* and *n*_0_ that give the finest-fitting and minimum deviation between the simulated and experimental curves are 3.010 ± 0.017 and (1.277 ± 0.043) × 10^6^ cm^−3^, respectively. The minimum FOM value for this peak is 0.547%, achieved after eight iterations, while the R-square of the fitting is 0.99825. The FOM value for the fitted peak is less than 1%, which implies that the fitted parameters are generating a theoretical curve matching very well with the experimental one. Also, the R-square value is very close to 1, which refers to the success of the fitting process. The residual curve for the present peak fluctuates between −0.02 and 0.02. A large portion of the residual curve is obtained from the tail of the glow peak in the range between 380 and 450 K, as seen in Figure 3b. Nonetheless, it does not affect the process to determine the trapping parameters of the peak.

The processes mentioned above in the case of the glow peak with *β* = 2 °C s^−1^ are repeated to fit and obtain the values of *b* and *n_o_* of the glow peaks with *β* = 0.5, 1, 3, 4 and 5 °C s^−1^. The values of *E* = 1.258 eV, *S*″ = 1.124 × 10^19^ s^−1^ are used with the initial guess of *b* = 1.001 and *n*_0_ = 1000 cm^−3^ for all the experimental curves. The value of *β* changes depending on the experimental curve. The best values of *b* and *n*_0_ that give the best fitting for the TL curves shown in Figure 1a are listed in Table 2. The number of iterations to achieve the best fitting is also listed up. The quality of the fitting was evaluated using R-square and FOM values. The obtained R-square and the calculated FOM values for the glow curves are given in Table 2. The FOM values of the fitted TL peaks, in general, less than 1% as a sign of the goodness of the fitting. The maximum and minimum values of FOM in Table 2 are 0.968% and 0.313% for the glow peaks with heating rates *β* = 0.5 and 5 °C s^−1^, respectively. This can be explained by the shift of the baseline of the experimental data that appeared in Figure 1a. This shift is maximum for *β* = 0.5 °C s^−1^ and minimum for *β* = 5 °C s^−1^. Further, the R-square values for all the glow peaks are very close to 1, which means the fitting accuracy is robust.

It is worth mentioning here that the TL glow curve with heating rate *β* = 2 °C s^−1^ was analyzed using the TPA method and the trap parameters were estimated to *b* = 3.024, *E* = 1.263 eV, *S*″ = 1.135 × 10^19^ s^−1^, and *n*_0_ = 1.352 × 10^6^. The results of the obtained parameters match very well with those obtained from the present method.

The values of *b* given in Table 2 are very similar for all glow peaks with different heating rates. Indeed, the shape of the glow peaks did not change significantly by changing the heating rates (Figure 1a). The values of *n*_0_ determined using the O-CFP and given in Table 2 are decreasing with increasing the heating rate from *β* = 0.5 °C s^−1^ to 5 °C s^−1^, which can explain the decrease of the TL intensity with increase the heating rate. Inherently, due to the decline of the initial populations of the electrons due to the thermal quenching. The residual of the fitted curves for different heating rates was fluctuating between −0.04 to 0.056 for *β* = 0.5 °C s^−1^ and −0.008 to 0.017 for *β* = 5 °C s^−1^. The trap parameters, given in Table 2 of the glow peaks shown in Figure 1a, will be used in the next section to anticipate the fading tendency of La_2_O_3_: Dy^3+^, Li^+^ TL peaks against the storage conditions, such as the storage time and temperature.

In the present work, the accuracy of the fitting and hence the trap parameters determination is measured using two different evaluation values, R-square and FOM. From the results shown in Table 2, the values of R-square and FOM can be considered as a good indicator for the fitting quality, and hence the trap parameters determinations. This can negate the assumption that using a mix of different methods might lead to a significant error in the obtained parameters.

### 3.2. The Fading of the La_2_O_3_: Dy^3+^, Li^+^ Glow Curves Signals

**Figure 4:**
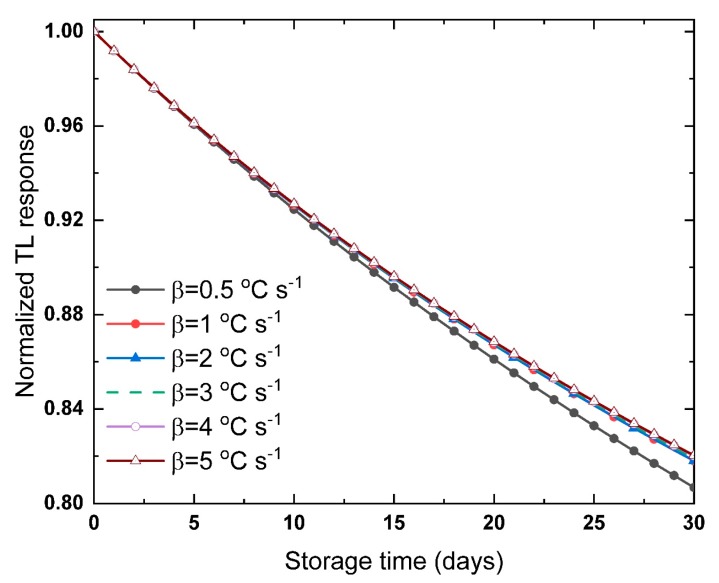
The fraction of TL signals for La_2_O_3_: Dy^3+^, Li^+^ recorded at different heating rates, estimated using Equation (23) and parameters given in Table 2, as a function of the storage time up to 30 days in 300 K storage temperature.

The stability of the TL signal against the storage conditions is considered a countermeasure of the capability of the luminescent material as a radiation dosimeter. Therefore, a better understanding of the fading of the TL signals is essential to justify the La_2_O_3_: Dy^3+^, Li^+,^ as a radiation dosimeter. The trap parameters are given in Table 2, with the values of *E* and *S*″, and are used in Equation (23) to examine the fading of the TL glow curves shown in Figure 1a. The response of the TL glow peaks, recorded at different heating rates, against the storage time is shown in Figure 4. Generally, the fading of the TL curves increases with increasing the period of storage and the storage temperature. Moreover, the shallow trap centers, with low activation energy values, have a high affinity to fade out in the room temperature. Moreover, the high values of the frequency factor, the probability of electrons to escape, leads to an increase in the TL signal fading with keeping the other conditions constant. Whenever the storage temperature is close to the *T_m_* of the glow peak, the thermal fading of the signal increase with the time. The values of *E* and *S*″ are constant for the TL signals shown in Figure 4, while, only the value of kinetics order is changing, assuming normalized TL signals *n/n*_0_. As shown in Figure 4, under the same storage temperature 300 K, and the same values of *E* and *S*″, the TL glow peaks are losing ~17% of the signal during the storage for 30 days. The depth of the trap centers of the glow peaks is quite deep, E = 1.258 eV, but the frequency factor is high, *S*″ = 1.124 × 10^19^ s^−1^. These two parameters affect the fading of the samples in a contradictory way. The change of *b* value, in Table 2, raises a variation of the TL fraction, as seen in Figure 4.

The small difference in the fading between the glow peaks is due to the difference in the probability of retrapping. In the first order model, the electron escape from the trap center will immediately recombine in a recombination center. On the other hand, in the second order model, the probability of retrapping is much higher, which means a delay in the light emitted from the TL signal. From here, one can understand the reason for the appearance of the second order peak broader than the first order, with lower TL intensity, even though having the same total area under the curve and same trap parameters. In the second order, if the electrons escape, it takes a longer time for recombining, and hence the TL light is emitted. An increase in *b* increases the probability of retrapping, and then introduces a delay of the emitted TL light. Therefore, a delay in the fading of the TL signal with increasing the time of storage must be expected. Hence, under the same conditions and same values of *E*, *S*″ of the glow peaks in Figure 1a, the peak with higher *b* value is expected to have lower fading, and vice versa, as seen in Figure 4. This suggests, from the fading point of view under the same conditions, the phosphor material with a higher *b* value is preferable as a radiation dosimeter.

The analysis of the trap parameters and the fading of the TL glow peaks of La_2_O_3_: Dy^3+^, Li^+^, revealed that the material could be used safely as a radiation dosimeter material. The predicted fraction of the TL signal, after 30 days at 300 K of irradiation, is >80% of its initial signal, which is high enough to conclude information about the irradiation process after one month.

### 3.3. Applications of TPA Method to Deconvolute and Determine Trap Parameters of La_2_O_3_: Eu^3+^, Li^+^ Glow Curves

The first aim of this section is to apply the TPA method on the complex TL glow curves of La_2_O_3_: Eu^3+^, Li^+^ appeared in Figure 1b, and then to determine the trap parameters of the individual peaks. By increasing the heating rate from 0.5 to 5 °C s^−1^, the curve position is shifted to a higher temperature while the maximum intensity is getting smaller (Figure 1b). The analysis of the TL curve recorded at *β* = 0.5 °C. s^−1^ will be illustrated in detail in the present section, while the other TL glow curves are identically treated, and only concluding results will be given for simplicity. It is seen from Figure 1b that the glow curve seems to consist of three main peaks, the highest temperature glow band with a maximum intensity at ~505 K, the more pronounced glow peak appeared at ~437 K, and the low-temperature band comes into view at 386 K. We will show that the glow curve includes four glow peaks. The flow of the deconvolution process and the trap parameter determination are given in the next paragraphs.

The separation process starts with the high-temperature peak, appearing at ~505 K, named Peak 1. Run−1: a set of three points *x*, *y*, and *z*, corresponding to three different temperatures on the descending part of the glow curve, are selected starting from *T_m_*. The values of (*T_x_*, *T_y_*, *T_z_*), and the corresponding intensities (*I_x_*, *I_y_*, *I_z_*), and the areas under the curve (*A_x_*, *A_y_*, *A_z_*), from the points until *T_f_*, are identified from the glow curve. By inserting these values in Equation (13), the order of kinetics *b* of Peak 1 can be calculated. In sequence, after calculating the value of *b*, the value of the activation energy *E* can be estimated using Equation (14) or (15). Hence, using *b*, *E*, *T_m,_* and *I_m_*, the values of *S* or *S*″ can be determined using Equations (16) and (17) for the first and general order, respectively. Eventually, the relative value of the initial concentration of the electrons can be estimated according to Equation (18).

**Figure 5:**
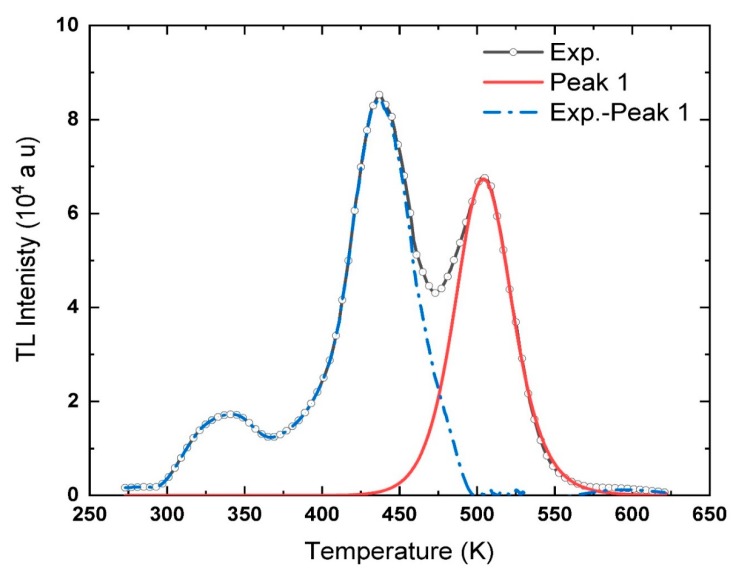
The experimental glow curve of La_2_O_3_: Eu^3+^ recorded at *β* = 0.5 °C s^−1^, plotted with the deconvoluted curve for Peak 1, and the residual glow curve after subtracting Peak 1.

The sequence mentioned above is repeated two times, Run-2 and Run-3, with a random order of the chosen temperatures from the descending portion of the glow curve. The contribution of the lower temperature peaks on the present peak was canceled through the calculations. That happened by avoiding using points with temperatures lower than *T_m_* during the process. The values used in Run−1, Run-2, and Run-3, with the calculated trap parameters, *b*, *E*, *S*″, and *n*_0_, of Peak 1 are listed in Table 3. The values of *T_m_* and the corresponding *I_m_* used in the calculations are given as well in Table 3. The average values of the trap parameters for Peak 1 are found equal to *b* = 1.867 ± 0.024, *E* = 1.625 ± 0.011 eV, *S*″ = (6.152 ± 0.074) ×10^14^ s^−1^ and *n*_0_ = (6.669 ± 0.054) ×10^6^ (a.u.). These parameters are used to simulate a theoretical peak using Equation (5). The experimental glow curve, i.e., black line with circles, is sketched with the simulated peak, red line, and the subtraction of Peak 1 from the experimental data, Exp.-Peak 1, and shown as a blue dashed line in Figure 5.

It can be seen from Figure 5 that the simulated peak is well-fitting the descending part of the experimental glow curve. The tail of the glow curve after ~560 K appears shooting from the fitted peak, which can be related to the fact that the baseline of the experimental data was not well calibrated. Peak 1 was separated from the complex glow curve, by applying the TPA method on the descending part of the glow curve. Also, the trap parameters of the peak were determined. Now part of the experimental curve, Exp.-Peak 1 in Figure 5, will be used in the next steps to determine the trap parameters of the lower temperature peak, appearing at ~437 K, Peak 2.

The same sequence used to deconvolute Peak 1 is applied to separate and determine the trap parameters of Peak 2. It is worth mentioning that the descending part can be used freely to calculate the trap parameters of the present peak, while to avoid the contribution of the lower temperature peaks, only 40% of the raising part is used in these calculations. Run−1: a random set of temperatures without any systematic relation was chosen. The values of the corresponding intensities and areas are identified from the glow curve and used to calculate *b* from Equation (13). In sequence, the trap parameters *E*, *S*, *S’*’ and *n*_0_ are estimated using Equations (14)–(18), respectively.

**Figure 6:**
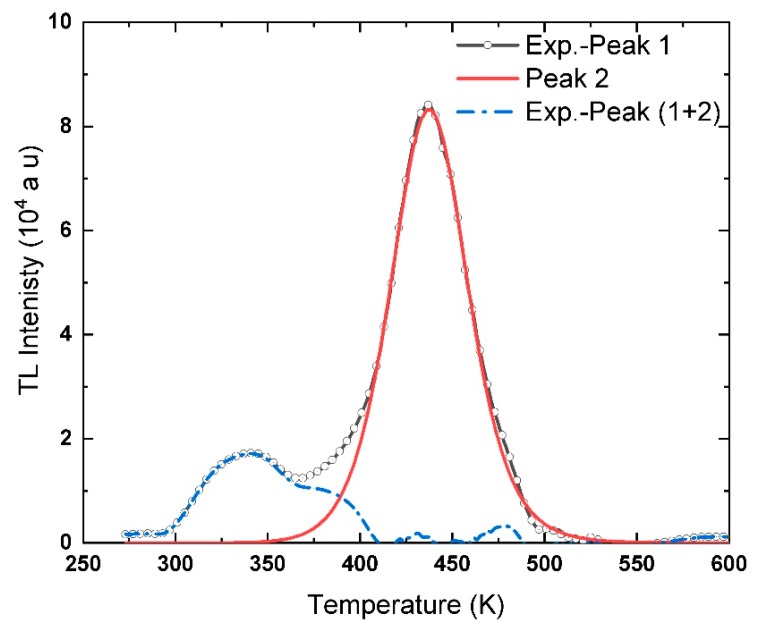
A part of the experimental curve, black line with circles, plotted with Peak 2, red line, and the subtraction of Peak 2 from the Exp.-Peak 1, dashed blue line.

Again, different sets of temperatures were used in Run-2 and Run-3 to determine the trap parameters of Peak 2. The points chosen from the Exp.-Peak 1 curve are listed in Table 3 with the values of the calculated trap parameters. The average values of the trap parameters for Peak 2 are *b* = 1.728 ± 0.016, *E* = 1.040 ± 0.012 eV, *S*″ = (2.828 ± 0.144) ×10^14^ s^−1^ and *n*_0_ = (9.225 ± 0.059) ×10^6^ (a.u.). Again, these values are used in Equation (5) to generate a theoretical curve for Peak 2. The theoretical curve is sketched in Figure 6 with Exp.-Peak 1. The theoretical peak is subtracted from the Exp.-Peak 1 curve and plotted in Figure 6 and named Exp.-Peak (1 + 2).

Obviously, from Figure 6, the theoretical peak, i.e., red line, fits very well with the experimental curve, black with circles, in most of the rising and descending parts of the curve. A small deviation between the experimental and the theoretical curve appeared frequently, is due to the accuracy deficiency of digitizing the curves. Regardless of this deviation, the trap parameters determination is not affected in general; only the error bar might be decreased with the residual curve. The residual of the TL glow curve after subtracting Peak 1 and Peak 2, dashed blue line in Figure 6, show a small peak at *T_m_* ~386 K, named Peak 3, in addition to the central peak ~337 °C, Peak 4. Only the present technique is capable of detecting such kind of hidden peaks, which is very hard to be observed from a general view of the TL glow curve since most of the deconvoluted programs and techniques use well-identified values of *T_m_* and *I_m_* as input to deconvolute the peaks [24,39,46,49,50]. In the present glow curve, Peak 3 is entirely blind between the pronounced peaks, Peak 2 and Peak 4.

**Figure 7:**
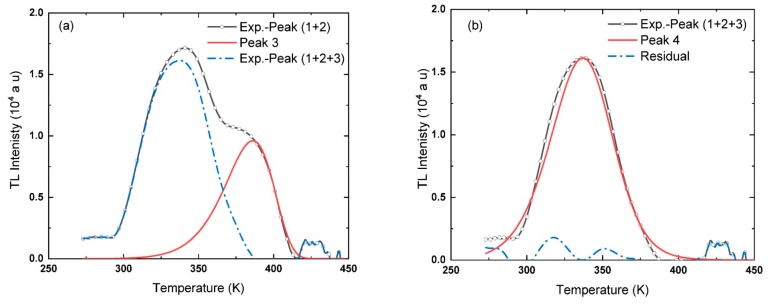
The separation of Peaks 3 (**a**) and Peak 4 (**b**) by applying the TPA method.

The same sequence used to deconvolute and determine the trap parameters of Peak 1 and 2 is used to analyze Peak 3 and 4. Starting with Peak 3, only the descending part of the Exp.-Peak (1 + 2), i.e., dashed blue line in Figure 6, is used to determine the trap parameters, owing to the overlap with Peak 4 in the raising part. Three sets of temperatures are used to determine the trap parameters of Peak 3. The values of these temperatures and corresponding intensities and areas in addition to the trap parameters are listed in Table 4. These parameters are used to simulate a shape for Peak 3 using Equation (5) and then plotted with Exp.-peak (1 + 2) in Figure 7a. Subtracting Peak 3 from Exp.-peak (1 + 2) gives a part of the experimental data, named Exp.-peak (1 + 2 + 3), blue dashed line in Figure 7a. The residual after subtracting Peak 3 is the leading curve of Peak 4.

In the last stage of this sequence, three sets of temperatures are chosen from the remaining part of the experimental curve. The distribution of the points may cover the whole curve, raising and descending portions. Nevertheless, around 20% of the rising and the descending parts were excluded to decrease the discrepancy of the calculated parameters. The values used to estimate *b*, *E*, *S*, *S’*’ and *n*_0_ of Peak 4 are given in Table 4. The theoretical peak, Peak 4, is plotted with Exp.-peak (1 + 2 + 3) in Figure 7b. The residual of the glow curve after subtracting Peak 4 is also depicted in Figure 7b.

**Figure 8:**
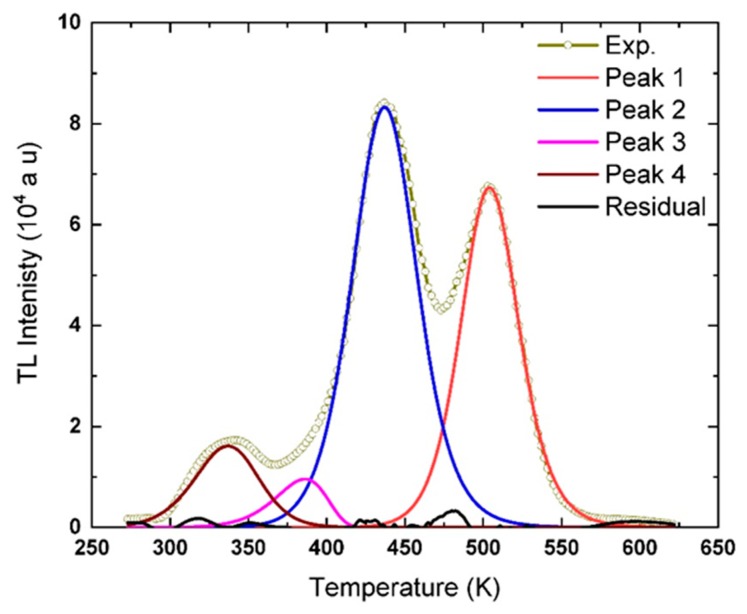
The experimental TL glow curve of La_2_O_3_: Eu^3+^, Li^+^ recorded at *β* = 0.5 °C s^−1^, and the deconvoluted peaks given in Table 5, the residual of the glow curve after subtracting the glow peaks are shown as well.

The analysis of the La_2_O_3_: Eu^3+^, Li^+^ recorded at *β* = 0.5 °C s^−1^ using the TPA method revealed that the glow curve consists of four peaks at ~505, ~ 437, ~386, and ~337 K. The trap parameters of peaks are determined, and the averages values are listed in Table 5. The averages values of the trap parameters are used to simulate the shape of glow peaks using Equation (5). The shape of the peaks and the experimental glow curve are depicted in Figure 8, and it appeared matching very well the experimental curve. The residual of the glow curve after subtracting the simulated peaks, Peak 1, 2, 3, and 4, is also charted in Figure 8. From the figure that the residual curve is not continuous and seems <0.01% of the experimental TL glow curve. The small fraction of the residual curve is one piece of evidence supporting the accuracy of the fitting method and the separation of the glow curve into the components of the glow peaks.

The TPA technique is used to perform the analysis of TL glow curves of La_2_O_3_: Eu^3+^, Li^+^ with *β* = 1, 2, 3, 4, and 5 °C s^−1^. The average values of the trap parameters of glow curves are summarized in Table 6, with the values of *T_m_* for every peak. Generally, when the heating rate increases, the maximum intensity of the peak decreases, and the peak position is shifting to the higher temperatures. The values of *I_m_* and *T_m_* for Peak 1 of the glow curves recorded at *β* = 0.5, 1, and 2 °C s^−1^, appeared in Figure 1b, are comfortable to be identified. However, the structure of the peak becomes smoother with the higher heating rates *β* = 3, 4, and 5 °C s^−1^. This makes identifying the peak position more laborious and time-consuming. The same tendency was observed for Peak 3 in all the glow curves shown in Figure 1b. At the same time, it is a good demonstration of the merits of using the TPA method. Such a tendency of the peaks is considered one of the significant obstacles of the earlier approaches to determine the trap parameters.

The value of the order of kinetics *b* is the cornerstone to determine the values of *E*, *S*, *S’*’ and *n*_0_ in the general order glow kinetics model. Therefore, several methods and expressions have been suggested and applied to determine the value of *b*. The symmetry factor *μ_g_* method to determine *b* is one of the most referenced methods in this filed [30]. The value of the symmetry factor is estimated based on the information of the peak shape, *µ_g_* = (*T*_2_ − *T_m_*)/(*T*_2_ −*T*_1_), where *T*_2_ > *T*_1_ are the temperatures at which the TL intensity is equal to half of *I_m_* for the peak. These temperatures, *T*_1_ and *T*_2_*,* should be located on raising and descending parts of the curve, respectively.

Obtaining these temperatures with reasonable accuracy for an isolated peak is considered an effortless task. While it becomes puzzling if two glow peaks are overlapped and infeasible for the complex glow curves [50,51,52,53,54]. In most cases of the overlapped TL glow curves, one side of the peak interferes with other peaks. For instance, the rising portion of Peak 1 of La_2_O_3_: Eu^3+^, Li^+^ TL glow curves is entirely hidden. Meanwhile, for Peak 3, the rising and descending parts of the peak are entirely overlapped with Peak 4 and Peak 2, respectively. On the other hand, the sequence of the TPA method gives the advantage to overcome the significant drawbacks over the different methods to determine *b*, such as the symmetry factor method. Other fitting methods and programs, such as the Gaussian functions, are invalid with glow curves obeying the general order kinetics curves.

Eventually, the TPA method has been applied successfully to analyze and determine the trap parameters of the TL glow curves of La_2_O_3_: Eu^3+^, Li^+^ recorded at *β* = 0.5, 1, 2, 3, 4, and 5 °C s^−1^. Four peaks were disclosed and separated, starting from the high-temperature peak. The effect of the overlapping between the peaks was eliminated through the sequence for separating the glow peaks. It is correct that the analysis of the TL glow curves using the first or second order models is critical to obtain the physical trap parameters; however, in many cases, these models are not satisfied to do the analysis of these TL glow curves. Here, the importance of using the general order kinetics equation to fill this gap. The main drawback of the present method is the difficulty in applying the technique on a highly complex TL glow curve, or the non-ending glow curves [26].

### 3.4. The Fading of the La_2_O_3_: Eu^3+^, Li^+^ Glow Curves

**Figure 9:**
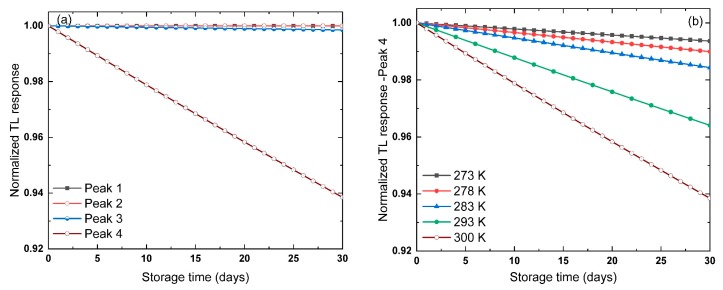
The TL response for La_2_O_3_Eu^3+^, Li^+^ recorded at *β* = 0.5 °C s^−1^, estimated using Equation (23) and parameters given in Table 5 as a function of storage time for 30 days (**a**) Peaks 1, 2, 3 and 4 at 300 K, (**b**) Peak 4 at different storage temperature 273, 278, 283, 293, and 300 K.

The fading of the TL glow curves of the La_2_O_3_: Eu^3+^, Li^+^, at different heating rates, is investigated to evaluate the possibility of using the material in radiation dosimeter applications. As the glow curves appeared in Figure 1b consists of four peaks. It is also essential to identify which peak is the one with high stability against the fading. The trap parameters for the TL glow curve with *β* = 0.5 °C s^−1^, given in Table 5, are used in Equation (23) to predict the tendency of the peaks against the storage conditions. Figure 9a shows the normalized TL response of the Peaks 1, 2, 3, and 4 as a function of storage time for 30 days at 300 K storage temperature. Approximately 6% of the TL signal of Peak 4 was faded out after 30 days. However, less than 0.5% from Peaks 1, 2, and 3 were faded out. This can be attributed to the low activation energy of the peak and the high-frequency factor, as seen in Table 5. Therefore, Peaks 1, 2, and 3 are more stable against fading compared to Peak 4.

The influence of the storage temperature on Peak 4 is also investigated using Equation (23) at different temperatures 273, 278, 283, 293, and 300 K. The decay of the TL signal as a function of temperature for Peak 4, for 30 days is shown in Figure 9b. As expected, when the storage temperature goes up, the fading level of Peak 4 following the tendency. The fraction of the faded-out TL signal is ranging from 0.8% at 273 K to ~6% at 300 K.

The fading of the TL glow peaks of La2O3: Eu3+, Li+ recorded at β = 0.5 °C s−1 is negligibly small, which suggests that the material can be employed as radiation dosimeter using all peaks included. Similar behavior was observed from the TL glow curves recorded at β = 1, 2, 3, 4, and 5 °C s−1. The results show that Peak 4 has a high fraction of the fading among all peaks of the glow curves with different heating rates. In the same sequence, the TL glow curve recorded at β = 5 °C s−1 shows the most significant fading ~16% of the signal at 300 K. The rest of peaks revealed different fading levels below 5%, even though this observation the La2O3: Eu3+, Li+ material seems to be a strong candidate for radiation dosimeter applications, from the fading point of view. In summary, the fading fraction of La2O3: Eu3+, Li+, and La2O3: Eu3+, Li+ is less than 20% of the initial signal. This suggests the stability of the materials against the fading is worth using for radiation dosimeter applications.

## 4. Conclusions

The trap parameters of the La_2_O_3_ recorded at different heating rates 0.5, 1, 2, 3, 4, and 5 °C s^−1^ after exposure to 5.12 Gy beta radiation were determined in this work. A non-linear curve fitting program named O-CFP, based on the general order kinetics equations and the outcomes of Hoogenstraaten’s Method, was developed and applied for La_2_O_3_: Dy^3+^, Li^+^ in this study. The trap parameters of the TL glow curves were determined using the O-CFP. The R-square and FOM values were used to evaluate the fitting quality. The three-points analysis technique was applied to separate and determine the trap parameters of the overlapped glow curves of La_2_O_3_: Eu^3+^, Li^+^. The method successfully deconvoluted the glow curves into four peaks, and then the trap parameters for the peaks were estimated in sequence starting from the high-temperature peak. The fading of the La_2_O_3_ activated with Dy^3+^ and Eu^3+^ against the storage conditions was investigated. The results revealed that ~17% of the TL signal of La_2_O_3_: Dy^3+^, Li^+^ is expected to fade out if stored at 300 K for 30 days. The TL signal of Peak 4, in case of La_2_O_3_: Eu^3+^, Li^+^ recorded at *β* = 0.5 °C s^−1^, the TL is expected to fade out with ~6% if it is stored under the same conditions, and 16% is expected for *β* = 5 °C s^−1^. The study suggests that La_2_O_3_: Eu^3+^, Li^+,^ and La_2_O_3_: Dy^3+^, Li^+^, are expected to show promising behavior if employed in radiation dosimeter applications.

## Figures and Tables

**Table 1:** The heating rates and the corresponding maximum temperatures for La_2_O_3_: Dy^3+^, Li^+^ TL glow curves given in Figure 1a.

***β* (°C s^−1^)**	0.5	1	2	3	4	5
***T_m_* (°C)**	41	46	50	53	55	57

**Table 2:** The best-fitting values of *b* and *n*_0_, obtained from the O-CFP for the TL glow curves presented in Figure 1a, the values of *E* = 1.258 eV, *S*″ = 1.124 × 10^19^ s^−1^ are used in the program. The corresponding heating rate, number of iterations, R-square, and FOM values also given.

*Β* (°C s^−1^)	b	*n_o_* × 10^6^ (cm^−3^)	FOM%	R-Square	Iterations
0.5	2.929 ± 0.013	4.837 ± 0.031	0.968	0.99972	13
1	3.011 ± 0.019	2.541 ± 0.096	0.636	0.99774	11
2	3.015 ± 0.017	1.277 ± 0.043	0.547	0.99825	8
3	3.093 ± 0.016	0.871 ± 0.027	0.497	0.99845	7
4	3.158 ± 0.015	0.669 ± 0.019	0.464	0.99873	8
5	3.174 ± 0.012	0.536 ± 0.014	0.313	0.99884	8

**Table 3:** The trap parameters of Peak 1 at ~505 K, and Peak 2 at ~437 K, calculated using the TPA method with the values of temperatures, intensities, and areas.

Peak 1	Run−1	Run-2	Run-3	Peak 2	Run−1	Run-2	Run-3
*T_x_* (K)	505	510	525	*T_x_* (K)	446	437	423
*T_y_* (K)	529	507	505	*T_y_* (K)	438	420	430
*T_z_* (K)	512	528	517	*T_z_* (K)	460	465	451
*I_x_* × 10^4^	6.757	6.532	3.965	*I_x_* × 10^4^	7.903	8.434	6.302
*I_y_* × 10^4^	3.021	6.689	6.757	*I_y_* × 10^4^	8.450	5.512	7.732
*I_z_* × 10^4^	6.366	3.270	5.750	*I_z_* × 10^4^	5.103	4.205	7.064
*A_x_* × 10^5^	1.777	1.441	0.612	*A_x_* × 10^6^	1.739	2.499	3.562
*A_y_* × 10^5^	0.475	1.641	1.777	*A_y_* × 10^6^	2.414	3.742	3.071
*A_z_* × 10^5^	1.315	0.507	1.015	*A_z_* × 10^6^	0.832	0.634	1.383
*I_m_* × 10^4^	6.757	6.757	6.757	*I_m_* × 10^4^	8.434	8.434	8.434
*T_m_*	505	505	505	*T_m_*	437	437	437
*b*	1.895	1.869	1.837	*b*	1.711	1.723	1.750
*E* (eV)	1.636	1.628	1.610	*E* (eV)	1.024	1.044	1.051
*S*″ × 10^14^	6.059	6.152	6.242	*S*″ × 10^10^	2.769	3.026	2.688
*n*_0_ × 10^6^	6.733	6.674	6.602	*n*_0_ × 10^6^	9.177	9.190	9.308

**Table 4:** The trap parameters estimated for Peak 3, left, and Peak 4, right, calculated using equations (13–18) with the values of temperatures, intensities, and areas used in the calculations.

Peak 3	Run−1	Run-2	Run-3	Peak 4	Run−1	Run-2	Run-3
*T_x_* (K)	400	386	388	*T_x_* (K)	325	313	345
*T_y_* (K)	386	395	401	*T_y_* (K)	310	349	354
*T_z_* (K)	393	399	397	*T_z_* (K)	318	360	310
*I_x_* × 10^3^	5.720	9.840	9.409	*I_x_* × 10^4^	1.459	0.925	1.583
*I_y_* × 10^3^	9.840	7.930	5.299	*I_y_* × 10^4^	0.792	1.4931	1.267
*I_z_* × 10^3^	8.421	6.170	7.131	*I_z_* × 10^4^	1.201	0.915	0.792
*A_x_* × 10^4^	0.364	1.432	1.279	*A_x_* × 10^6^	6.107	7.823	2.879
*A_y_* × 10^4^	1.432	0.665	0.272	*A_y_* × 10^6^	8.023	2.279	1.798
*A_z_* × 10^4^	0.808	0.405	0.501	*A_z_* × 10^6^	7.288	1.082	8.023
*I_m_* × 10^3^	9.840	9.840	9.840	*I_m_* × 10^4^	1.632	1.632	1.632
*T_m_*	386	386	386	*T_m_*	337	337	337
*b*	0.965	0.952	0.921	*b*	1.577	1.479	1.576
*E* (eV)	0.740	0.750	0.764	*E* (eV)	0.584	0.602	0.609
*S*″ × 10^8^	1.336	1.843	1.735	*S*″ × 10^7^	2.868	2.806	3.387
*n*_o_ × 10^6^	8.417	7.925	7.925	*n*_0_ × 10^6^	1.704	1.645	1.691

**Table 5:** The average values of the trap parameters estimated for the TL glow curve of La_2_O_3_: Eu^3+^, Li^+^ recorded at *β* = 0.5 °C s^−1^, with the corresponding maximum temperatures.

Parameters	Peak 1	Peak 2	Peak 3	Peak 4
*T_m_*	505	437	386	337
*b*	1.867 ± 0.024	1.728 ± 0.016	0.924 ± 0.049	1.544 ±.046
*E* (eV)	1.625 ± 0.021	1.040 ± 0.019	0.754 ± 0.034	0.604 ± 0.023
*S*″ (s^−1^)	(6.152 ± 0.074) × 10^14^	(2.828 ± 0.144) × 10^10^	(2.305 ± 0.103) × 10^8^	(3.021 ± 0.261) × 10^7^
*n_o_* (a.u)	(6.669 ± 0.054) × 10^6^	(9.225 ± 0.059) × 10^6^	(8.099 ± 0.342) × 10^5^	(1.680 ± 0.256) × 10^6^

**Table 6:** The average values of the trap parameters for La_2_O_3_: Eu^3+^, Li^+^ recorded at *β* = 1, 2, 3, 4, and 5 °C s^−1^ calculated using Equations (13)–(18) and corresponding maximum temperatures of the peaks.

Parameters	Peak 1	Peak 2	Peak 3	Peak 4
*β (*°C s^−1^)	1	1	1	1
*T_m_*	515	447	387	344
*b*	1.788 ± 0.046	1.808 ± 0.019	1.367 ± 0.033	1.486 ± 0.057
*E* (eV)	1.641 ± 0.019	1.037 ± 0.024	0.835 ± 0.021	0.608 ± 0.017
*S*″ (s^−1^)	(7.969 ± 0.304) × 10^14^	(2.834 ± 0.250) × 10^10^	(4.979 ± 0.085) ×10^9^	(4.801 ± 0.429) × 10^7^
*n*_0_ (a.u.)	(2.881 ± 0.011) × 10^6^	(4.793 ± 0.071) × 10^6^	(4.535 ± 0.059) × 10^5^	(8.378 ± 0.230) × 10^5^
*β* (°C s^−1^)	2	2	2	2
*T_m_*	522	458	391	347
*b*	1.832 ± 0.043	1.863 ± 0.036	1.452 ± 0.074	1.397 ± 0.084
*E* (eV)	1.623 ± 0.037	1.041 ± 0.029	0.800 ± 0.066	0.617 ± 0.043
*S*″ (s^−1^)	(6.234 ± 0.165) ×10^14^	(3.111 ± 0.267) × 10^10^	(8.148 ± 0.571) × 10^9^	(8.272 ± 0.681) × 10^7^
*n*_0_ (a.u.)	(1.339 ± 0.019) × 10^6^	(2.397 ± 0.021) × 10^6^	(3.350 ± 0.110) ×10^5^	(4.044 ± 0.134) × 10^5^
*β (*°C s^−1^)	3	3	3	3
*T_m_*	529	464	395	350
*b*	1.886 ± 0.068	1.881 ± 0.067	1.491 ± 0.059	1.413 ± 0.052
*E* (eV)	1.640 ± 0.053	1.039 ± 0.054	0.831 ± 0.071	0.609 ± 0.047
*S*″ (s^−1^)	(8.565 ± 0.723) × 10^14^	(3.313 ± 0.547) × 10^10^	(7.239 ± 0.393) ×10^9^	(9.527 ± 0.269) × 10^7^
*n*_0_ (a.u.)	(8.536 ± 0.183) × 10^5^	(1.627 ± 0.012) × 10^6^	(1.971 ± 0.078) × 10^5^	(3.049 ± 0.052) × 10^5^
*β (*°C s^−1^)	4	4	4	4
*T_m_*	532	469	401	354
*b*	1.954 ± 0.028	1.756 ± 0.031	1.484 ± 0.014	1.426 ± 0.072
*E* (eV)	1.631 ± 0.025	1.045 ± 0.023	0.827 ± 0.037	0.621 ± 0.031
*S*″ (s^−1^)	(6.806 ± 0.121) × 10^14^	(3.762 ± 0.621) × 10^10^	(6.694 ± 0.864) × 10^9^	(1.281 ± 0.145) × 10^8^
*n*_0_ (a.u)	(6.379 ± 0.059) × 10^5^	(1.166 ± 0.019) × 10^6^	(1.397 ± 0.014) × 10^5^	(2.269 ± 0.061) × 10^5^
*β* (°C s^−1^)	5	5	5	5
*T_m_*	536	475	406	358
*b*	2.025 ± 0.012	1.783 ± 0.023	1.485 ± 0.032	1.415 ± 0.049
*E* (eV)	1.641 ± 0.027	1.048 ± 0.041	0.815 ± 0.039	0.611 ± 0.045
*S*″ (s^−1^)	(8.509 ± 0.457) × 10^14^	(3.432 ± 0.205) × 10^10^	(7.000 ± 0.979) × 10^9^	(1.090 ± 0.149) × 10^8^
*n*_0_ (a.u.)	(5.296 ± 0.027) × 10^5^	(9.478 ± 0.061) × 10^5^	(1.206 ± 0.019) × 10^5^	(1.876 ± 0.046) × 10^5^

**Figure A1:**
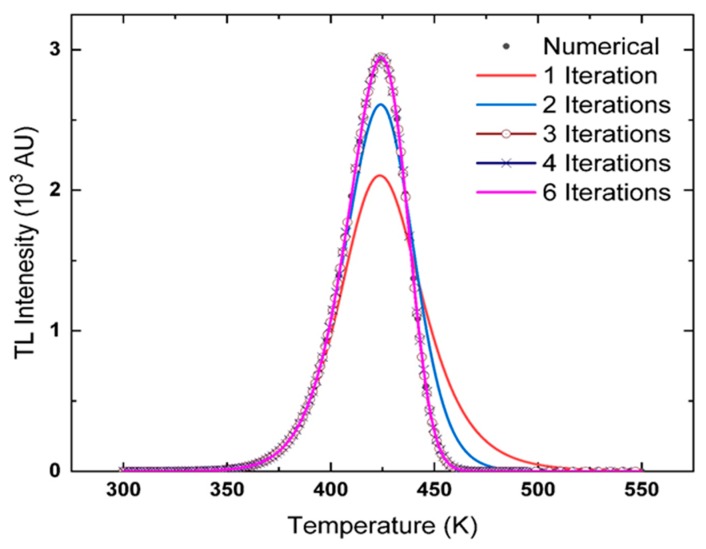
The steps by using O-CFP to obtain the best values of *b* and *n*_0_ and in Example 1.

**Figure A2:**
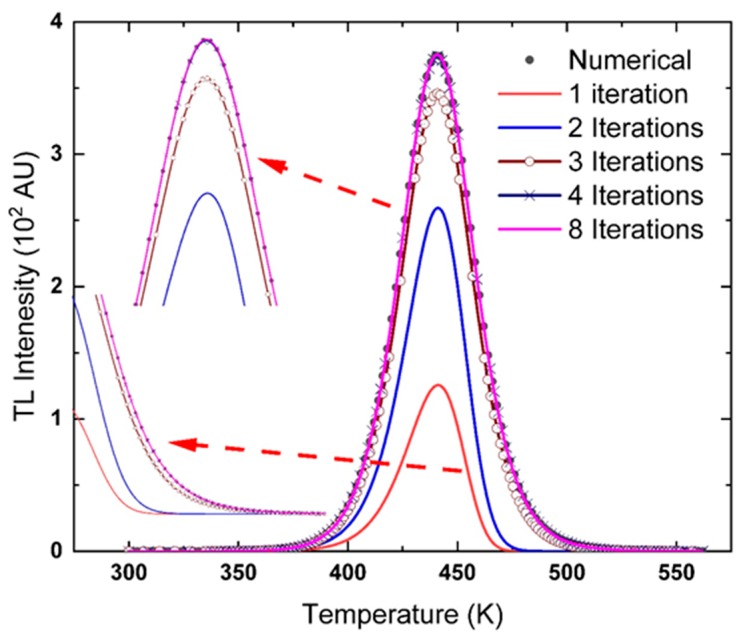
The steps by using O-CFP to obtain the best values of *b* and *n*_0_ and for Example 2.

## Appendix A

### The Evaluations of the O-CFP for Curve-Fitting and the Calculation of b and n_0_

This merely constitutes a self-consistency test stating essentially that no errors were made in the calculation steps of our program. The evaluation of the O-CFP for obtaining *b* and *n*_0_ is demonstrated here by taking some numerically generated TL glow peaks. Initially, Equation (5) was used to create numerical peaks using a set of parameters including *b*, *n*_0_, *E*, *S*″ and *β*. These curves are inserted into the O-CFP (i.e., the intensity and the temperature) instead of experimental data. The values of *E*, *S*″ and *β* used to generate the peak are inserted to the O-CFP as invariable parameters. The first guess for *b* and *n*_0_ is added into the program with the possibility of changing by examine R-square and FOM values. The first iteration using the above values (*b*, *n*_0_, *E*, *S″,* and *β*) is used to generate a theoretical peak. This peak is compared with the numerical peak. Based on the R-square and FOM values, the program will suggest the new parameters of the *b* and *n*_0_ for the second iteration. These steps continue until a minimum value of FOM, and the maximum value of R-square is obtained. Two examples confirm the accuracy of using the O-CFP for getting *b* and *n*_0_ of a general-order glow peak and are presented in the next paragraphs.

**Example** **1.**
*Firstly, Equation (5) is used to generate a numerical TL glow peak using the selected values of trap parameters, such as: b = 1.111, n_0_ = 1.111 × 10^5^ cm^−3^, E = 1.111 eV, S″ = 1.111 × 10^12^ s^−1^ and β = 1 °C s^−1^, respectively. The numerical peak generated using the above parameters is shown in Figure A1. Secondly, insert the data of the numerical peak and the values E = 1.111 eV, S″ = 1.111 × 10^12^ s^−1,^ and β = 1 °C s^−1^ into the O-CFP to make the appropriate analysis for obtaining the resultant values of b and n_0_. The guessing values of b = 1.001 and n_0_ = 1000 cm^−3^ are provided to the program to generate the first simulated peak, iteration 1 in Figure A1. In general, the simulated peak generated using the first guess is far away from the numerical peak. The shape is mainly dependent on the values of the first guess. Based on the deviation between the simulated and numerical peaks, the O-CFP suggests the second guess. The suggested values, in this case, after one iteration, are found equal to b = 1.98681 and n_0_ = 1.105937 × 10^5^ cm^−3^. The next step from the program is to repeat the same process several times, iterations. Take into consideration the R-square and FOM values, by changing the values of b and n_0_ until a minimum deviation between the simulated and numerical peaks occurs. The minimum deviation, in this case, has occurred after six iterations with b = 1.11084 and n_0_ = 1.111078906 × 10^5^ cm^−3^, the values of R-square and FOM are 1 and 0.0189%, respectively. Table A1 shows the resulting values of b and n_0_ after different iterations of the analysis with the values of FOM and R-square, while Figure A1 shows the growth fitted curves with the experimental one.*

**Table A1:** The parameters and the sequential steps for analyzing the numerically generated glow peak appearing in Figure A1, according to the O-CFP, with increasing the number of iterations.

No of Iterations	Input Parameters				Resulting Parameters			
	b	*n*_0_ (cm^−3^)	E (eV)	S″ ×10^12^	b	*n*_0_ (cm^−3^)	FOM %	R-Square
1	1.001	1000	1.111	1.111	1.98681	110593.713	35.577	0.88491
2	1.98681	110593.713	1.111	1.111	1.46691	114373.675	14.263	0.9741
3	1.46691	114373.675	1.111	1.111	1.10297	110875.051	0.3246	0.99998
4	1.10297	110875.051	1.111	1.111	1.11072	111073.192	0.0241	1
6	1.11072	111073.192	1.111	1.111	1.11084	111078.906	0.0189	1

**Example** **2.**
*The second example is to illustrate the possible change of fitting steps if one selected initial guessing values of n_0_ and/or b higher than the real values of n_0_ and/or b. A numerically glow peak is generated according to Equation (5), using the parameters: b = 1.777, n_0_ = 5555 cm^−3^, E = 1.333 eV, S″ = 4.0 × 10^12^ s^−1^ and β = 3 °C s^−1^, while the first guess in the present case is b = 2.000, n_0_ = 1000 cm^−3^. The numerical peak generated using the above parameters is shown in Figure A2. The same sequence used in Example is repeated in the present case. The minimum deviation obtained after eight iterations with b = 1.7769 and n_0_ = 5554.416 cm^−3^, the values of R-square and FOM are 1 and 0.0105%, respectively. Table A2, shows the resulting values of b and n_o_ after different iterations of the analysis with the values of FOM and R-square, while Figure A2 shows the growth fitted curves with the experimental one.*

*Thus, we found the calculated kinetic parameters of TL glow curves coincide precisely with the predetermined ones. Moreover, the FOM values have a tiny percent, which means we reached the minimum deviation between the numerical and simulated peaks.*

**Table A2:** The input and output parameters given in Example 2, for the first guess, is higher than the real value of the *b*.

No of Iterations	Input Parameters				Resulting Parameters			
	b	*n*_0_ (cm^−3^)	E (eV)	S″ × 10^14^	b	*n*_0_ (cm^−3^)	FOM %	R-Square
1	2.000	1000	1.333	4.0	1.0911	1419.619	291.279	0.31647
2	1.0911	1419.619	1.333	4.0	1.1841	3060.372	81.503	0.77973
3	1.1841	3060.372	1.333	4.0	1.7044	4995.686	11.192	0.98911
4	1.7044	4995.686	1.333	4.0	1.7768	5543.408	0.2086	0.99999
8	1.7768	5543.408	1.333	4.0	1.7769	5554.416	0.0105	1

In conclusion, it is found that the O-CFP fitting program can safely fit the numerically calculated glow curves.
